# Pancreatitis with use of new diabetic medications: a real-world data study using the post-marketing FDA adverse event reporting system (FAERS) database

**DOI:** 10.3389/fphar.2024.1364110

**Published:** 2024-05-27

**Authors:** Khalidah A. Alenzi, Deemah Alsuhaibani, Bader Batarfi, Thamir M. Alshammari

**Affiliations:** ^1^ Society of Pharmacovigilance, Jeddah, Saudi Arabia; ^2^ Transformation, Planning, and Business Development Department, Tabuk Health Cluster, Tabuk, Saudi Arabia; ^3^ Pharmaceutical Care Department, Medical Services for Armed Forces, Ministry of Defense, Riyadh, Saudi Arabia; ^4^ Medication Safety Research Chair, King Saud University, Riyadh, Saudi Arabia; ^5^ College of Pharmacy, Almaarefa University, Riyadh, Saudi Arabia

**Keywords:** drug-induced, glucagon-like peptide-1, inhibitors of dipeptidyl peptidase 4 (DPP-4), pancreatitis, sodium-glucose transport protein 2 (SGLT2) inhibitors

## Abstract

**Background:** Pancreatitis is characterized by inflammation of the pancreas and significantly affects quality of life. Less than 5% of pancreatitis cases are drug-induced, but recent evidence suggests a substantial risk associated with glucagon-like peptide-1 receptor agonists (GLP-1 RAs). The aim of this study was to compare the risk of developing pancreatitis between those using GLP-1 RAs and those using sodium-glucose transport protein 2 (SGLT2) inhibitors and dipeptidyl peptidase 4 (DPP-4) inhibitors.

**Methods:** This study was done using the FDA Adverse Event Reporting System (FAERS) database from 2019 to 2021. This database contains information from diverse submissions from healthcare providers, patients, and manufacturers. To ensure fairness and accuracy, the risk of pancreatitis associated with other hypoglycemic agents (SGLT2 inhibitors and DPP-4 inhibitors) was also investigated. Traditional and Bayesian statistical analysis methods were used to identify disproportionate statistics and included the reporting odds ratio (ROR), proportional reporting ratio (PRR), empirical Bayes geometric mean (EBGM), and information component (IC). A drug–adverse-event combination that met the criteria of all four indices was deemed a signal.

**Results: **The analysis of 2,313 pancreatitis reports linked to hypoglycemic agents revealed a predominant association with GLP-1 RA (70.2%) compared to DPP-4 inhibitors (15%) and SGLT2 (14.7%). Most of these reports involved female patients (50.4%), and the highest incidence occurred in those over 50 years old (38.4%). Additionally, 17.7% of the reports were associated with serious events. The ROR was significant for the risk of pancreatitis when using DPP-4 (13.2, 95% confidence interval (CI) 11.84-14.70), while the ROR for GLP-1 was 9.65 (95% CI 9.17-10.16). The EBGM was highest with DPP-4 (12.25), followed by GLP-1 (8.64), while IC was highest with DPP-4 inhibitors (3.61). Liraglutide had the greatest association with pancreatitis among the GLP-1 RAs (ROR: 6.83, 95% CI 6.60-7.07).

**Conclusion:** The findings show that pancreatitis has a strong link with DPP-4 inhibitors and GPL1 agonists, which pose a greater risk. Among the GLP-1 agonist medications, liraglutide has been found to have an association with pancreatitis.

## 1 Introduction

Glucagon-like peptide-1 receptor agonists (GLP-1 RAs) are a standard class of medications that are used to treat type 2 diabetes mellitus (T2DM) (Collins and Costello, 2024) These medications mimic the actions of a hormone naturally found in the intestines, glucagon-like peptide-1 (GLP-1), which regulates blood sugar levels by increasing insulin secretion and decreasing glucagon release. GLP-1 RAs not only improve glycemic control but also have cardioprotective and renoprotective effects and are effective for weight loss in patients with obesity with and without diabetes (Seksaria, et al., 2024).

A broad spectrum of GLP-1 RAs has been approved by the Federal Drug Administration (FDA) to treat T2DM, including exenatide, liraglutide, albiglutide, dulaglutide, lixisenatide, and semaglutide (Liu, et al., 2022). GLP-1 RAs or sodium-dependent glucose transporter 2 (SGLT2) inhibitors are often recommended for patients with T2DM who have atherosclerotic cardiovascular disease (ASCVD) or indicators of high risk for it due to the cardiovascular benefits they provide (American Diabetes Association, 2021). Although these agonists are generally deemed safe and efficacious (Nauck et al., 2021), it is crucial to note that they have potential risks. One of them is pancreatitis, a condition that results in pancreas inflammation.

The pancreas plays a crucial role in the human body and has essential endocrine and exocrine functions. The endocrine functions involve the production of hormones that regulate blood sugar levels and glandular secretion, while the exocrine functions are related to digestion. Inflammation of the pancreas can lead to severe complications, with hospitalization being necessary in some cases (Wei, et al., 2020).

Pancreatitis has several risk factors, including gallstones, alcohol abuse, smoking, hypertriglyceridemia, and some medications. Furthermore, patients with diabetes mellitus (DM) have a higher chance of developing pancreatitis compared with those who have no DM (Zhang, et al., 2023). Also, there is evidence suggesting that DM is also a risk factor for pancreatic cancer (Shen, et al., 2023).

A cohort study conducted in 2022 evaluated the onset of acute pancreatitis and pancreatic cancer in patients with T2DM who used SGLT2 inhibitors compared to those who used dipeptidyl peptidase-4 (DPP-4) inhibitors. The results showed that SGLT2 inhibitors had a significantly lower acute pancreatitis rate than DPP-4 inhibitors. SGLT2 inhibitors were also associated with lower risks of acute pancreatitis and pancreatic cancer (Chou et al., 2023).

Another study assessed 524, 510, and 40 spontaneous reports of pancreatitis from the FDA Adverse Event Reporting System (FAERS) database, VigiBase, and CARD databases, respectively, to determine the potential association between SGLT2 inhibitors and pancreatitis. The analysis discovered a correlation between SGLT2 inhibitors and pancreatitis, with empagliflozin presenting the highest risk. Case reports supported the findings, emphasizing the importance of early diagnosis through physical examination and laboratory parameters (Palapra, et al., 2022).

In contrast, a meta-analysis of 11 studies comprising 55,921 patients confirmed that GLP-1 RA and DPP-4 inhibitors medications do not pose a risk of pancreatic cancer. Furthermore, DPP-4 inhibitor treatment notably increased the risk of acute pancreatitis by a staggering 75% (Lee et al., 2019). Recent meta-analyses also indicate that GLP-1 RA does not increase the likelihood of pancreatitis and pancreatic cancer compared to a placebo and other treatments (Pinto, et al., 2019).

Research on the link between GLP-1 agonists and pancreatitis has yielded conflicting results. Some studies indicate a potential association between GLP-1 agonists and an increased risk of pancreatitis (Abd El Aziz, et al., 2020; Liu, et al., 2022). According to a retrospective analysis of the United States FDA database, it is evident that from 2004 to 2009, exenatide treatment resulted in a tenfold increase in reported cases of pancreatitis compared to other therapies, while others have not found a significant connection (Elashoff et al., 2011). In contrast, recent meta-analyses that included large-scale cardiovascular outcome trials (CVOTs) suggest that GLP-1 RAs do not significantly increase the risk of acute pancreatitis or pancreatic cancer in patients with T2DM (Cao, et al., 2020). However, previous meta-analyses did not include these CVOTs, highlighting a gap in the literature (Monami et al., 2017).

Additionally, GLP-1 RAs’ labels warn about acute pancreatitis and impose an obligation upon doctors to inform patients about its symptoms. A systematic review examined long-term, placebo-controlled, randomized controlled clinical trials on GLP-1 RA in which acute pancreatitis was a predefined adverse event. The study found no evidence that treatment with GLP-1 RA increases the risk of AP in patients with T2DM (Storgaard, et al., 2017).

The influence of GLP-1 RA on the pancreas, including increasing insulin production and stimulating GLP-1 receptors in pancreatic tissues, may contribute to pancreatitis (Chen, et al., 2022). It is imperative to differentiate between acute pancreatitis, which is often reversible with proper medical management, and chronic pancreatitis, which is a syndrome that results in chronic pain, exocrine and endocrine pancreatic insufficiency, reduced quality of life, and a shorter life expectancy. Chronic pancreatitis is caused by repetitive episodes of pancreatic inflammation that lead to extensive fibrotic tissue replacement, and no curative treatment is available (Hart and Conwell, 2020; Ashraf, et al., 2021). The present study explored the correlation between using GLP-1 RA and the likelihood of developing pancreatitis. It was compared with two other hypoglycemic agent categories: SGLT2 inhibitors and DPP-4 inhibitors.

## 2 Methods

### 2.1 Study design and settings

This observational pharmacovigilance study used the FAERS database, which is a publicly available post-marketing safety surveillance database. The database can be freely accessed at https://open.fda.gov/data/faers/. The FDA publishes the FAERS datasets quarterly (every 3 months). It plays a pivotal role in capturing reports of adverse events, medication errors, and product-quality complaints from diverse contributors such as health professionals, pharmaceutical manufacturers, lawyers, and individual patients. It is important to note that FAERS is a US database that receives adverse-event reports worldwide. Due to its large size and global coverage, this open database is suitable for analyzing spontaneous data reporting. However, pharmacovigilance databases store anonymized information, ensuring data privacy is not compromised.

The FAERS database includes eight types of files: demographic and administrative information (DEMO), drug information (DRUG), adverse events (REAC), patient outcomes (OUTC), report sources (RPSR), start and end dates for reported drugs (THER), indications for use (INDI), and invalid reports (DELETED). All files record “primaryid” and “caseid” variables, which means that information about patients and AEs can be obtained by linking these variables in all files. DRUG and THER files record “drug_seq” variables, which means that information about drug use and therapy can be obtained by linking “drug_seq” variables in these two files (Poluzzi, et al., 2012).

### 2.2 Data collection and filtration

A thorough analysis of data gathered from the FAERS database was done to shed light on the potential link between the use of GLP-1 RA agonists and the risk of pancreatitis. The analysis was done using data from 2019 to 2021 from meticulously analyzed reports submitted to the FDA by healthcare providers, patients, and manufacturers. For fairness and accuracy, we also examined the risk of pancreatitis associated with other hypoglycemic agents: SGLT2 inhibitors and DPP-4 inhibitors (Supplement A).

The outcome of interest was pancreatitis, which was searched using preferred term (pt) “pancreatitis”. To minimize bias, we included the primary suspected drug (PS) that is associated with the outcome of interest in the analyses. Duplicate reports were excluded using the primary identification number (primaryid) along with the event date (event_dt) and “pt”. PS was used to ensure that there were no duplicate reports.

### 2.3 Statistical analyses

A 2 × 2 contingency table was used to perform data mining for four metrics. The four four components (cells) of the 2 × 2 tables were labeled “a,” “b,” “c,” and “d,” where “a” represents the number of reports of cases (outcome of interest) for the studied drugs, and “b” is the number of reports of non-cases (no outcome of interest, adverse events) for those drugs. Furthermore, “c” represents the number of reported cases for other medications, and “d” represents the number of reports of non-cases for all other medications (Alsuhaibani, et al., 2023). Poluzzi et al. (2012) provides an explanation of the mathematical equations.

This research applied a blend of traditional and Bayesian statistical analysis methods to detect potential safety concerns related to drug combinations and adverse events. This dual-method approach enhanced the rigor of the analysis and helped to avoid incorrect findings.

The reporting odds ratio (ROR) served as a key analytical tool for comparing the probability of a specific drug causing an adverse event to all other drugs in the database. A higher ROR suggests a possible correlation between the drug and the adverse event. The proportional reporting ratio (PRR) was introduced to compare the reports for a specific combination of drugs and adverse events to those for all other combinations. A higher PRR suggests a potential indication for that combination.

The study also incorporated the empirical Bayes geometric mean (EBGM) and information component (IC) in the Bayesian analysis. The EBGM measures the estimated strength associated between a drug and an adverse event. Considering the underlying frequency of the event in the population provides a threshold for detecting signal patterns. The IC measures the disproportionality of specific combinations of drugs and adverse events compared to all other combination trends in the database. This multi-faceted statistical approach facilitated thorough and accurate signal detection. More detailed on these four analyses are explained eelsewhere in more detail (Poluzzi, et al., 2012).

To ensure the reliability of the findings, the signals considered significant had to fulfill criteria across all four indices. This approach ensures that only reliable and consistent signals are recognized and minimizes the chances of false associations.

The data mining tools had different thresholds based on the metrics used. For example, for ROR, any value more than one was considered a safety signal associated with the outcome of interest. As for PRR, the value was more than two, while for EBGM, the threshold was two or more. For IC, any value more than 0 was considered a safety signal (Jokinen, et al., 2019). In addition to these thresholds, reports counts for ROR and PRR of ≥2 and ≥3 were required, respectively. For EBGM and IC, the number of reports should be more than 0 (i.e., 1 and above) (Jokinen, et al., 2019,. All statistical analyses were done using the statistical software R (version 4.2.2) and RStudio (version 2022.12.0 + 353).

## 3 Results

**FIGURE 1:**
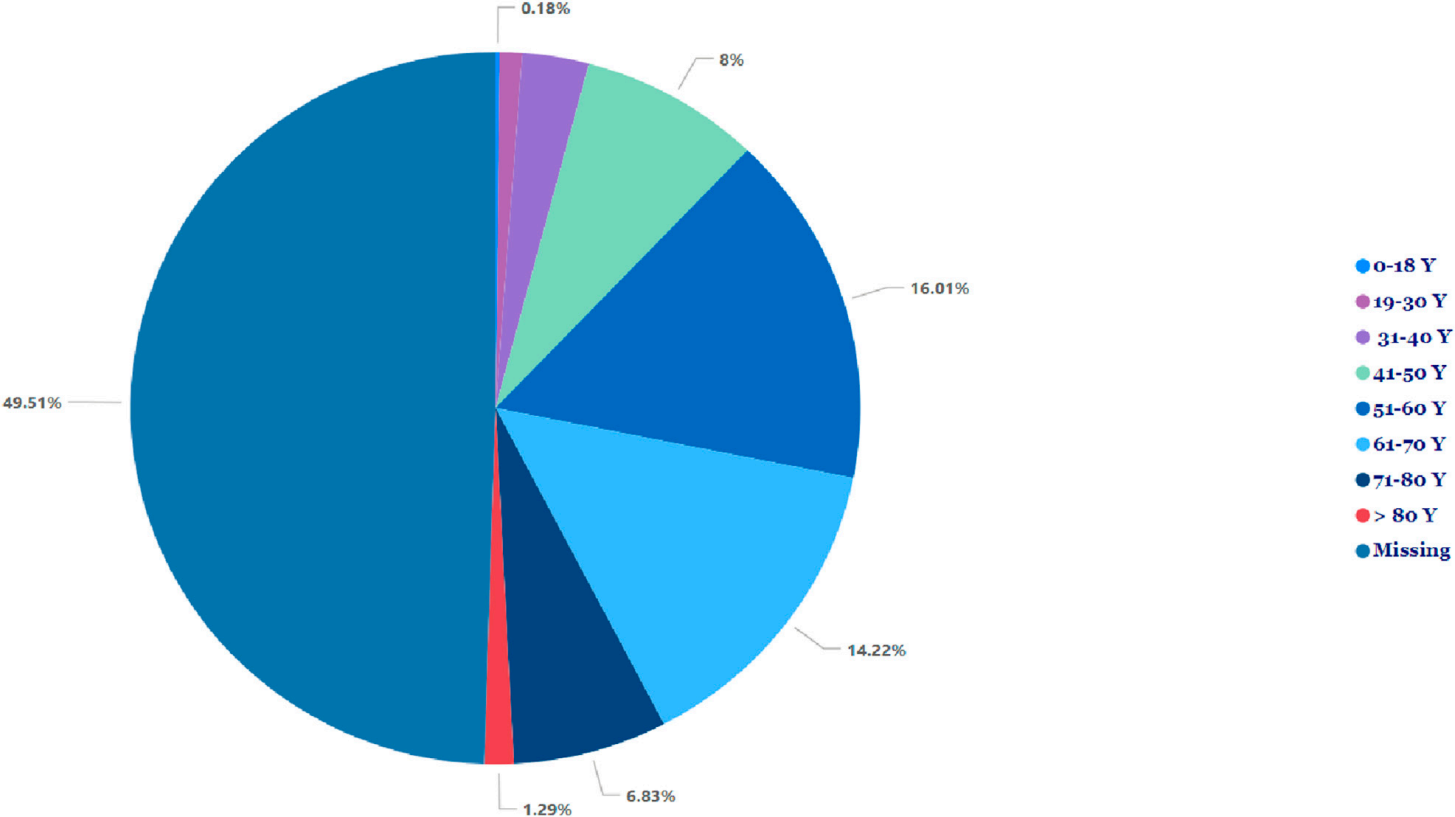
Incidence of pancreatitis associated with glucagon-like peptide-1 receptor agonist across different age groups.

**FIGURE 2:**
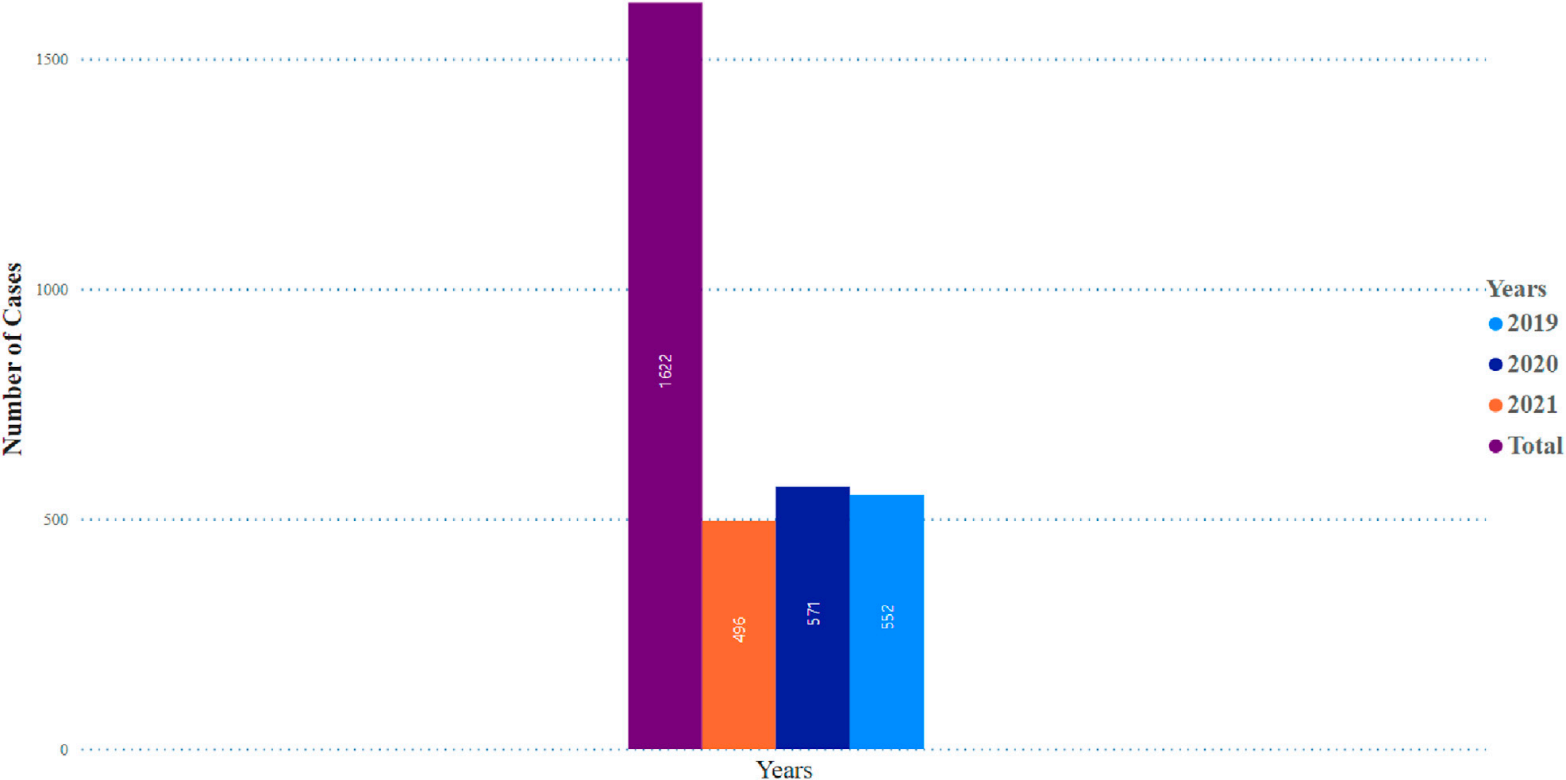
Pancreatitis incidence observed in glocagon-like peptide-1 receptor agonist users over a 3 years period.

Table 1 illustrates no significant difference in the frequency of pancreatitis reports between females (n = 705, 50.4%) and males (n = 692, 49.5%). The cases of pancreatitis associated with GLP-1 RA varied between age groups, with cases peaking in those between 51 and 60 years old, closely followed by the age range of 61–70 years (Figure 1). The youngest age group, 0–18 years, had the lowest occurrence rate of pancreatitis. In 2019, an increase in reported cases was observed, with 552 cases, peaking at 571 in 2020, followed by a slight decrease in 2021 to 496 cases (Figure 2).

**TABLE 1:** The occurrence rate of pancreatitis linked with glucagon-like peptide-1 receptor agonists for both males and females.

Sex	Frequency	Percent	Cumulative	Cumulative
			Frequency	Percent
Female	705	50.4	705	50.4
Male	692	49.5	1,397	99.91
*Frequency Missing = 226				

**FIGURE 3:**
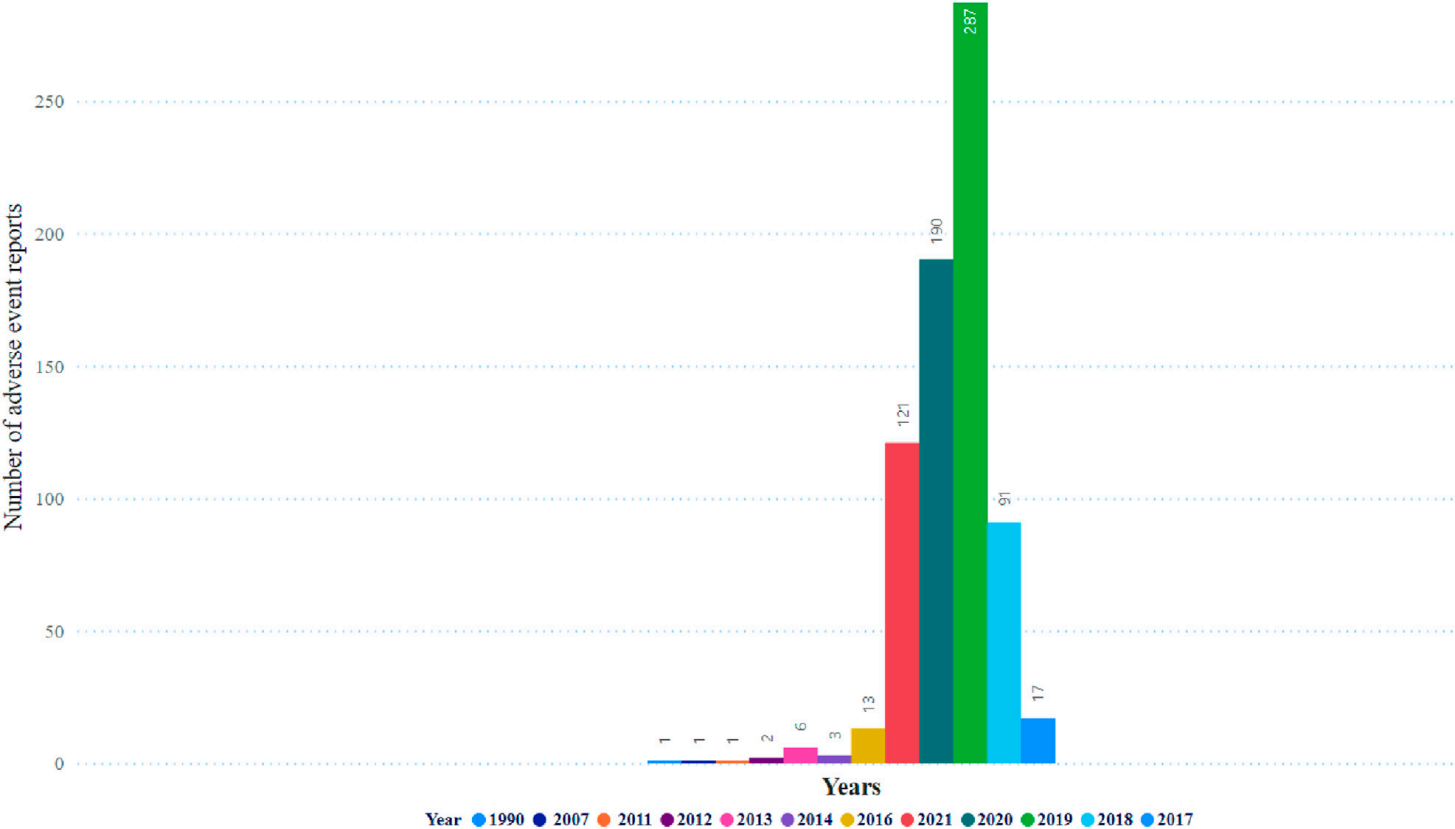
Distribution of glucagon-like peptide-1 antaginist adverse event reports over the years.

**FIGURE 4:**
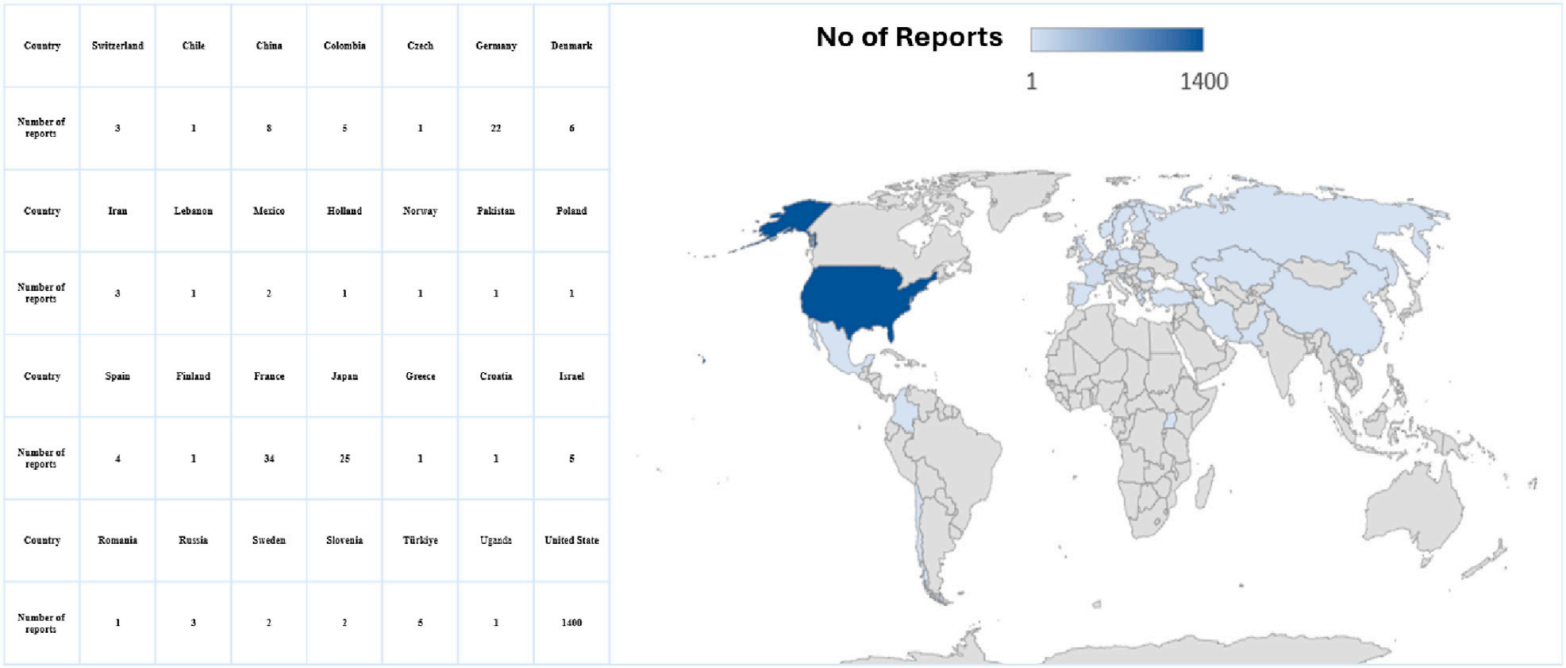
Forecast global density distribution of pancreatitis cases associated with glucagon-like peptide-1 receptor agonists.

Figure 3 displays the distribution of events, and the highest occurrence was 287 events. Subsequently, 190 events occurred in 2019 and 2020, respectively. Table 2 shows details of pancreatitis cases associated with GLP-1 RA drugs, including 12 fatalities, four disabilities, 208 hospitalizations, and 12 life-threatening cases, as well as one case requiring immediate intervention to prevent irreversible harm. The United States had the most reported cases (1,400 cases), as shown in Figure 4. Table 3 shows the safety risks of pancreatitis events for GLP-1 RA agonists, DPP-4 inhibitors, and SGLT2 inhibitors. Several statistical indices were used for accurate comparisons. The data show a difference in the number of reported pancreatitis cases for each drug class. For instance, GLP-1 RAs had 1,624 cases, DPP-4 inhibitors had 348 cases, and SGLT2 inhibitors had 341 cases. The ROR for GLP-1 RAs was 9.65 with a 95% confidence interval (CI) of 9.17–10.16. Conversely, DPP-4 inhibitors had a higher PRR of 12.67 (95% CI: 11.37-14.12) with ROR of 13.20 (11.84-14.70).

**TABLE 2:** Outcomes of pancreatitis associated with the use of GLP-1 RA.

DE	DS	HO	LT	OT	RI
12	4	208	12	287	1

DE, death; DS, disability; H, hospitalization; LT, Life-threatening; RI, Required Intervention to Prevent Permanent Impairment/Damage.

**TABLE 3:** Risk of pancreatitis events associated with GLP-1 Ras, DPP-4 inhibitors and SGLT2 inhibitors.

Drug class	Pancreatitis events	ROR (95% CIs)	PRR (95% CIs)	EBGM	IC
GLP-1 RAs	1,624	9.65 (9.17-10.16)	9.40 (8.92-9.90)	8.64	3.11
DPP-4 inhibitors	348	13.20 (11.84-14.70)	12.67 (11.37-14.12)	12.25	3.61
SGLT2 inhibitors	341	3.67 (3.29-4.09)	3.63 (3.26-4.05)	3.57	1.84

DPP-4, Dipeptidyl Peptidase-4; EBGM, empirical bayes geometric mean; GLP-1, RAs, Glucagon-like peptide-1, receptor agonists; SGLT2, Sodium-Glucose Cotransporter-2; PRR, proportional reporting ratio; ROR, reporting odds ratio.

SGLT2 inhibitors had an EBGM of 3.57, indicating an signal the drug class and an unfavorable event considering its frequency in the population. A higher IC value was reported for DPP-4 inhibitors (3.61) along with a higher ROR and PRR, indicating a possible signal with pancreatitis events. Table 3 shows that there is a clear relationship between pancreatitis and GLP-1 RAs. The ROR for pancreatitis was high for liraglutide at 6.83 (95% CI: 6.60-7.07). Semaglutide also had a high ROR of 5.69 (95% CI: 5.45-5.84). In contrast, for pancreatitis, dulaglutide had a relatively low ROR of 2.71 (95% CI: 2.63-2.79) (Table 4). It is vital to highlight that ROR values greater than 1 compared to the reference drug exenatide signifies a higher risk of pancreatitis for these drugs.

**TABLE 4:** Association between the signal of pancreatitis among GLP-1 RAs.

Drug	ROR^a^	(95% CIs)
Dulaglitide	2.71	2.63–2.79
Semaglutide	5.69	5.45–5.84
Liraglutide	6.83	6.60–7.07

ROR, reporting odds ratio.

^a^ Reference drug is exenatide.

## 4 Discussion

This study assessed the relationship of pancreatitis with GLP-1 RAs as well as DPP-4 inhibitors and SGLT2 inhibitors, which many studies consider old drugs compared to GLP-1 RAs. GLP-1 RAs were associated with the development of acute pancreatitis. Several pharmacovigilance and database studies have been conducted, and the results have needed to be consistent.

Our study results found that the risk of pancreatitis is almost similar among males and females. This suggests that the risk of pancreatitis was due to the studied medications. As for the risk of pancreatitis in diabetic patients of different genders, the literature found that there is no predominant gender in developing pancreatitis because the risk might be higher among males because of alcohol consumption, while the risk is higher among females, especially for gallstones (Weiss, et al., 2019).

The risk of pancreatitis in our study occurred more in higher age groups, i.e., between 51 to 60 and 61–70 years old. The risk of developing pancreatitis is expected to increase with a higher age group because usually type 2 diabetes develops after the age of 40, and these medications are commonly used in these age groups. Our results were aligned with the study conducted by Antonio Gonzalez-Perez et al. (Gonzalez-Perez et al., 2010). On the other hand, the youngest group (i.e., <18 years old) has a lower risk because those patients are usually with type 1 diabetes and are mainly treated with insulin.

The peak of pancreatitis events was in 2019, and it started to decrease in 2020 and 2021. This might be due to the impact of the coronavirus 2019 (COVID-19) pandemic that affected the healthcare systems in many countries, including access to medications, less reporting by both healthcare professionals and patients, and health institutions and regulatory bodies focusing more on the COVID-19-used medications. Later, the vaccines used, especially during the pandemic peak, were in 2020 and 2021. Most of the reports in our study are from the US because FAERS is a US database established by the FDA.

A comprehensive study also used data from the FAERS database and analyzed 71,515 records of GLP-1 RA monotherapy from 2005 to 2021. These records included 16,350 pairs of GLP-1 RA and important medical events. The study identified five significant disproportionality signals in the system organ class (SOC) categories: gastrointestinal disorders, investigations, metabolism, nutrition disorders, neoplasms, benign/malignant, and hepatobiliary disorders. The most common adverse events reported were pancreatitis, nausea, and weight decrease.

A comparison of our results with the literature reveals a complex landscape. One study identified significant disproportionality signals for gastrointestinal disorders related to GLP-1 RAs, which is consistent with our observations of more frequent reports of pancreatitis (Wu, et al., 2022)Such findings necessitate a closer examination of the underlying biological mechanisms. For example, the drugs could potentially lead to an increased rate of pancreatic enzyme production and secretion, which could induce pancreatic inflammation (Meier and Nauck, 2014).

A study was conducted using the French Pharmacovigilance Database to evaluate the incidence of pancreatitis in patients who were administered GLP-1 analogs and DPP-4 inhibitors. The mean age of patients who developed pancreatitis while on incretin-based drugs was found to be 60.4 ± 11.4 years, with 59.0 ± 10.2 years for GLP-1 agonists and 61.5 ± 12.2 years for DPP-4 inhibitors. The male gender accounted for 58% of the pancreatitis cases associated with incretin-based drugs. GLP-1 agonists and DPP-4 inhibitors were the sole antihyperglycemic agents reported in 46.6% and 25.4% of the cases, respectively. The hospitalization rate was notably high (89.8%), and fatal outcomes were observed in 2.1% of the reported cases (Faillie, et al., 2014).

A study conducted in France using the French Pharmacovigilance Database discovered that GLP-1 analogs and DPP-4 inhibitors were linked with most cases of pancreatitis (up to 55% and 37.41% of cases, respectively). The reports also demonstrated that males had a higher incidence of pancreatitis, accounting for 58% of cases, with an average age of 60.4 ± 11.4 years. Furthermore, the study revealed that 89.8% of the reported cases resulted in hospitalization, and 2.1% of the cases had fatal outcomes (Faillie, et al., 2014).

A recent cohort study conducted in Hong Kong investigated the incidence of acute pancreatitis in patients with T2DM who were administered either SGLT2 inhibitors or DPP-4 inhibitors between 2015 and 2020. The study included 31,609 patients, of whom 6,479 (20.49%) received SGLT2 inhibitor treatment, while 25,130 (70.50%) received DPP-4 inhibitor treatment. After careful matching, the results showed that patients who received SGLT2 inhibitor treatment had a significantly lower rate of acute pancreatitis than those who received DPP-4 inhibitors. Furthermore, the study also revealed that patients who were administered SGLT2 inhibitors had a considerably lower risk of developing pancreatic cancer than those who received DPP-4 inhibitors. The findings were confirmed using different propensity-score approaches and competing risk models, indicating consistency and reliability of the results (Chou, et al., 2023).

Our findings align with a previous study that used FAERS data from 2004 to 2009. The study revealed that the GLP-1 agonist drugs exenatide and sitagliptin were associated with a tenfold increase in reported pancreatitis events compared to other antidiabetic medications. The statistical analysis showed an odds ratio (OR) of 9.99 with a 95% CI of 7.26–14.1 (Elashoff, et al., 2011).

A recent meta-analysis of several CVOTs investigated the effects of GLP-1 RAs and DPP-4 inhibitors on the incidence of acute pancreatitis compared to a placebo or active comparators. The analysis found no significant increase in acute pancreatitis sin patients treated with GLP-1 RAs (N = 55,932; OR 1.05; 95% CI 0.77–1.42; *p* = 0.77). In contrast, patients treated with DPP-4 inhibitors showed a significant increase in acute pancreatitis in both placebo-controlled trials (N = 47, 714; OR, 1.81; 95% CI, 1.21–2.70; *p* = 0.04) and trials that compared DPP-4 inhibitors with a placebo or active comparators (N = 53,747; OR 1.54; 95% CI 1.08–2.18; *p* = 0.02) (Singh, et al., 2020).

Based on a pooled analysis of phase III clinical trials, it was found that GLP-1 RAs may pose a slightly elevated risk of pancreatitis (38 events, 17,775 patient-years of exposure) compared to the alternative treatment (9 events, 5,863 patient-years of exposure; OR: 1.39; 95% CI 0.67-2.88). It is essential to consider this information when making treatment decisions (Meier and Nauck, 2014). A comprehensive meta-analysis revealed that there was no significant difference in the risk of acute pancreatitis associated with the group receiving GLP-1 agonists *versus* the placebo group (Peto OR [95% CI] 1.05 [0.78–1.40], *p* = 0.76 and 1.12 [0.77–1.63], *p* = 0.56, respectively) (Cao, et al., 2020).

Furthermore, another meta-analysis provided evidence that contradicts the notion of an increased risk of acute pancreatitis in individuals treated with GLP-1 agonists (Monami, et al., 2014). A systematic review and meta-analysis of 60 studies included 55 randomized controlled trials (*n* = 33,350), and it was determined that GLP-1 agonists do not pose an increased risk of pancreatitis compared to controls. The data showed an OR of 1.05 with a 95% CI of 0.37–2.94 (Li, et al., 2014).

A recent study revealed that the occurrence of pancreatitis in diabetic patients can be attributed to a combination of various factors, including the disease itself, patient-related factors, and the administration of therapeutic agents. Additional factors, such as obesity, alcohol consumption, and smoking, can also increase the risk of pancreatitis, irrespective of drug therapy. The study also analyzed 17 studies involving over 100,000 participants and found that the use of GLP-1 agonists did not significantly affect the occurrence of pancreatitis and pancreatic cancer compared to using a placebo. The overall risk ratio (RR) for pancreatitis was 0.96, and the 95% CI ranged from 0.31 to 3.00, while the RR for pancreatic cancer was 1.10, and its 95% CI ranged from 0.31 to 4.10 (Zhang, et al., 2022).

Similarly, the use of DPP-4 inhibitors did not increase the risk of pancreatitis or pancreatic cancer. The overall RR for pancreatitis was 1.60, and the 95% CI ranged from 0.25 to 11.00, while the overall RR for pancreatic cancer was 0.79, and the 95% CI ranged from 0.26 to 2.40. The study also ranked lixisenatide and saxagliptin as the safest drugs in comparison to other drugs based on probability (Zhang, et al., 2022).

The disproportionality analyses in this study showed that liraglutide had the highest signal with pancreatitis (ROR: 6.83, 95% CI 6.60-7.07). A systematic review of case reports found that liraglutide was among the most frequently used medications in pancreatitis cases reported (Wolfe, et al., 2020). However, it is essential to note that a retrospective study using the FAERS database revealed that among all antidiabetic medications associated with pancreatitis, only exenatide and sitagliptin are significantly correlated with a disproportionality signal (ROR 1.76, [95% CI 1.61–1.92] and ROR 1.86 [95% CI 1.55–2.24]) and ROR 1.86 [95% CI 1.55–2.24], respectively) (Raschi et al., 2013). A case report showed that a morbidly obese elderly woman with T2DM who was treated with liraglutide experienced acute pancreatitis and cholestatic jaundice. The symptoms resolved clinically and biochemically once the liraglutide treatment was stopped (Farooqui, et al., 2019).

Our results with respect to the risk of pancreatitis with semaglutide and liraglutide support a study done by Marso et al. They included 3,297 patients with T2DM on a standard-care regimen. The study participants were divided into two groups, one receiving once-weekly semaglutide (0.5 or 1.0 mg) and one receiving a placebo. The results showed that patients who received semaglutide had a reduced risk of developing acute pancreatitis compared to the placebo group. Specifically, only nine patients treated with semaglutide experienced acute pancreatitis, while 12 patients in the placebo group were affected. These findings suggest that semaglutide may be a safer alternative to liraglutide for patients with T2DM who are at risk of developing pancreatitis (Marso, et al., 2016). Out of the 3,183 patients who took part in the PIONEER 6 study, it was found that one patient who received semaglutide treatment had an episode of acute pancreatitis. Three patients who received placebo treatment reported the same condition (Husain, et al., 2019).

It should be noted that the present study has certain limitations. For instance, the FAERS tool used for detecting post-marketing safety signals relies on spontaneous reporting, which may not always provide data of the highest quality. Moreover, the tool does not require proof of a causal relationship for submitted reports, making it difficult to draw definitive conclusions from the observed associations in the database.

It is also essential to remember that all signal detection can only suggest a statistical correlation. Further investigation and research are required to determine whether there is a real causal relationship. Finally, several other confounding factors may not be captured in the FAERS database, such as disease severity, comorbidities, and concurrent medications. Despite these limitations, it is worth noting that the FAERS database is still an important tool that the FDA uses for post-marketing surveillance.

Furthermore, it is also difficult to make conclusions about the causal relationships because there might be confounders such as the severity of DM and the occurrence of pancreatic cancers, among others. In addition, it is important to remember that the data collected in these studies are observational, meaning that factors other than GLP-1 RA therapy could have influenced the results. For instance, patients with diabetes who receive GLP-1 RA therapy might also have other risk factors for pancreatitis, such as obesity, taking other medications, or having diabetes for an extended period (Funch, et., 2014). Also, our study was conducted between 2019 and 2021; therefore, the results only reflect this investigation period. In addition, we used the term pancreatitis as the outcome of interest, utilizing the preferred term, which is general and not specific.

### 4.1 Implications

Our investigation into GLP-1 RAs has yielded findings with profound implications for public health and clinical practice. As T2DM becomes increasingly prevalent and GLP-1 RAs continue to be a mainstay treatment, there arises an imperative for augmented patient education and more vigilant drug safety surveillance systems to address the risk of medication-induced pancreatitis preemptively. The American Diabetes Association already highlights the prudence required in prescribing and advocating GLP-1 RAs. For heightened caution in patients predisposed to pancreatitis, this underscores the necessity for intensified scrutiny of pancreatitis symptoms in patients receiving GLP-1 RA treatment. The considerable number of adverse event reports related to GLP-1 RAs in our study signals urgency for a critical assessment of existing monitoring protocols and the need for their enhancement to facilitate prompt detection and intervention.

Methodologically, while the FAERS database is instrumental for post-marketing safety signal detection, it presents inherent limitations due to its reliance on voluntary reporting, which can introduce bias and does not substantiate causation. Thus, while the database significantly contributes to the generalizability of our findings, they should be interpreted with caution given that they primarily indicate statistical correlations. Dedicated research is essential to discern a concrete causal relationship, and this need is further accentuated by the potential for confounding variables not accounted for in the FAERS database, including the disease’s severity, comorbidities, and concurrent medication regimens.

The evolving landscape of drug safety and the nuances revealed by our study necessitate that healthcare policies and clinical guidelines be adaptable to these new insights. Updating clinical guidelines to integrate stringent monitoring for early signs of pancreatitis in patients administered GLP-1 RAs is paramount. Moreover, refining the standards for reporting adverse drug reactions is critical to bolstering the precision of post-marketing surveillance.

In summary, while the therapeutic benefits of GLP-1RAs in managing T2DM are indubitable, our findings serve as a call for healthcare professionals to balance efficacy with safety judiciously. The associated risk of pancreatitis calls for a conservative, patient-centric approach in diabetes management prioritizing informed consent and meticulous monitoring for adverse effects to mitigate potential harm.

## 5 Conclusion

According to the study’s findings, there is a strong link between pancreatitis and DPP-4 inhibitors and GPL1 agonists, which pose a greater risk. Healthcare providers must weigh the risks and benefits of prescribing GLP-1 agonists, DPP-4 inhibitors, and SGLT2 inhibitors to patients with pancreatitis, particularly those with a history of pancreatic disorders. It is essential to consider the potential for adverse effects, such as pancreatitis and the development of pancreatic cancer. Therefore, clinicians must take a careful and individualized approach when prescribing these agents for this patient population. By doing so, they can help ensure that patients receive the most appropriate and effective treatment for their condition while minimizing the risk of harm.

## Data Availability

The raw data supporting the conclusion of this article will be made available by the authors, without undue reservation.
