# Supplementary material for: Pancreatitis with use of new diabetic medications: a real-world data study using the post-marketing FDA adverse event reporting system (FAERS) database
Source: Front Pharmacol. 2024 May 27;15:1364110. doi: 10.3389/fphar.2024.1364110 (PMC11163090; doi:10.3389/fphar.2024.1364110)
Supplement: Supplementary file 1 [file DataSheet1.PDF]

Supplement A

Explaining the data management

1. Pharmacological classification: Dipeptidyl peptidase-4 (DPP-4) inhibitors	
Generic Name	Brand Name
Alogliptin	Nesina<br>Vipidia<br>Vipdomet<br>Kazano (combination with metformin)
Linagliptin	Tradjenta<br>Trajenta<br>Linagliptin<br>Glyxambi(combination with empagliflozin)
Saxagliptin	Onglyza<br>Kombiglyze (combination with metformin)
Sitagliptin	Januvia<br>Tesavel<br>Xelevia<br>Ristaben

2. Pharmacological classification : Glucagon-like peptide-1 receptor agonists (GLP-1 agonists)	
Generic Name	Brand Name
Albiglutide	Tanzeum
Dulaglutide	Trulicity
Exenatide	Byetta<br>Bydureon
liraglutide	Victoza<br>Saxenda
Semaglutide	Ozempic<br>Wegovy

3. Pharmacological classification : Sodium-glucose cotransporter 2 (SGLT2) inhibitors	
Generic Name	Brand Name
Canagliflozin	Invokana<br>Invokamet (combination with metformin)<br>Sulisent<br>Prominad
Dapagliflozin	Farxiga<br>Forxiga<br>Xigduo (combination of dapagliflozin with metformin)<br>Qtern(combination of dapagliflozin with saxagliptin)
Ertugliflozin	Steglatro<br>Segluromet (combination with metformin)<br>Steglujan (combination with sitagliptin)
Empagliflozin	Jardiance<br>Synjardy<br>Trijardy(Linagliptin combination with empagliflozin and metformin)
